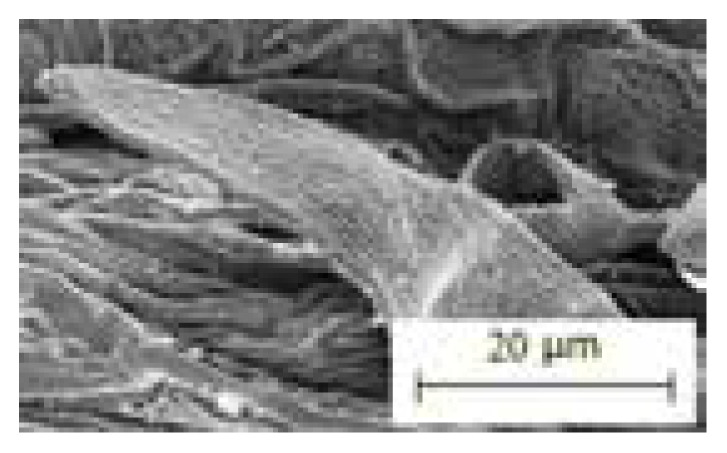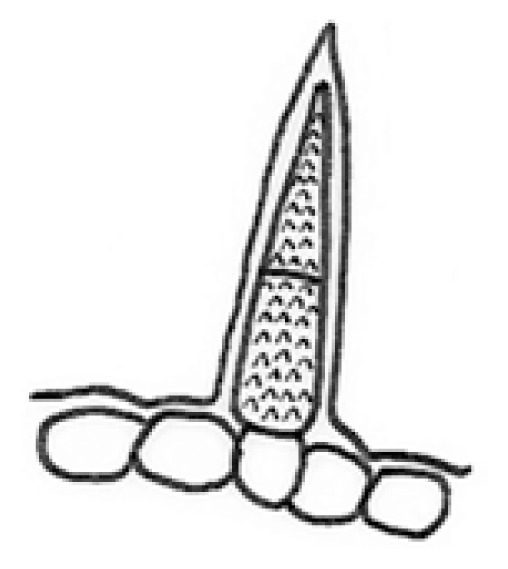# Comparative Leaf Anatomy and Micromorphology of *Thunbergia erecta* (Benth.) T. Anderson and *Thunbergia laurifolia* Lindl. in Peninsular Malaysia

**DOI:** 10.21315/tlsr2022.33.1.7

**Published:** 2022-03-31

**Authors:** Siti Maisarah Zakaria, Che Nurul Aini Che Amri, Noraini Talip, Amirul Aiman Ahmad Juhari, Mohamad Ruzi Abdul Rahman, Ahmad Fitri Zohari, Mohd Norfaizal Ghazalli, Nordahlia Abdullah Siam, Rozilawati Shahari

**Affiliations:** 1 Department of Plant Science, Kulliyyah of Science, International Islamic University Malaysia, Jalan Sultan Ahmad Shah, 25200 Kuantan, Pahang, Malaysia; 2 School of Environment and Natural Resource Sciences, Faculty of Science and Technology, Universiti Kebangsaan Malaysia, 43600 Bangi, Selangor, Malaysia; 3 Department of Environment, Faculty of Forestry and Environment, Universiti Putra Malaysia, 43400 UPM Serdang, Selangor, Malaysia; 4 Programme of Resource Utilisation and Agrobiodiversity Conservation, Agrobiodiversity and Environment Research Centre, 43400 MARDI Serdang, Selangor, Malaysia; 5 Forest Product, Forest Research Institute Malaysia, 52109 Kepong, Selangor, Malaysia

**Keywords:** Leaf Anatomy, Leaf Micromorphology, Taxonomic Significance, *Thunbergia*, Anatomi Daun, Mikromorfologi Daun, Nilai Taksonomi, *Thunbergia*

## Abstract

Comparative leaf anatomy and micromorphology study was carried out on two selected species from the genus *Thunbergia* Retz. of Acanthaceae subfamily Thunbergioideae. These two investigated species were *T. erecta* and *T. laurifolia* from Peninsular Malaysia. The leaf anatomical study involve several methods such as cross-section using sliding microtome on the petioles, midribs, lamina and marginal, leaf epidermal peeling, leaf clearing and observation under a light microscope. The leaf micromorphology method involve the observation under a scanning electron microscope (SEM). This study aimed to investigate the taxonomic value of leaf anatomy and micromorphology characteristics of genus *Thunbergia*. The results have shown that there were five common characteristics present in both species studied and several variable characters that might be useful for species differentiation of *T. erecta* and *T. laurifolia*. The five common characteristics recorded were the presence of raphide, sinuous anticlinal walls, diacytic stomata, majority opened and minority closed venation in lamina and the presence of peltate glandular (unicellular terminal) trichome. The variable characteristics included were petiole, and marginal outlines, types of vascular bundles, the presence of druse, marginal venation, stomata occurrence, types of wax, cuticular sculpturing and types of trichomes. In conclusion, findings in this study showed that leaf anatomical and micromorphological characteristics possessed taxonomic value that can be used in the species identification for the genus *Thunbergia* specifically for *T. erecta* and *T. laurifolia*.

## Abstract (fr)

Kajian perbandingan ciri anatomi dan mikromorfologi daun telah dijalankan ke atas dua spesies terpilih dalam genus *Thunbergia* Retz. (Acanthaceae) subfamili Thunbergioideae. Dua spesies yang telah dikaji ialah *T. erecta* dan *T. laurifolia* dari Semenanjung Malaysia. Kajian anatomi daun ini melibatkan beberapa kaedah seperti kaedah hirisan dengan mikrotom gelongsor pada bahagian petiol, tulang daun, lamina dan tepi daun, kaedah siasatan epidermis, kaedah penjernihan dan cerapan di bawah mikroskop cahaya. Kajian mikromorfologi melibatkan cerapan di bawah mikroskop imbasan elektron. Kajian ini bertujuan untuk melihat nilai taksonomi ciri anatomi dan mikromorfologi daun dalam genus *Thunbergia*. Hasil kajian menunjukkan terdapat lima ciri sepunya ke atas spesies yang dikaji dan beberapa ciri variasi yang boleh digunakan untuk pembezaan di antara *T. erecta* dan *T. laurifolia*. Lima ciri sepunya yang telah direkodkan ialah kehadiran rafid, dinding antiklinal sinuous, stomata diasitik, peruratan lamina daun majoriti terbuka dan minoriti tertutup serta kehadiran trikom kelenjar peltat (terminal unisel). Ciri variasi termasuklah bentuk petiol dan tepi daun, jenis tisu vaskular, kehadiran drus, kehadiran stomata, peruratan tepi daun, jenis lilin, ukiran kutikel dan jenis trikom. Kesimpulannya, hasil kajian menunjukkan ciri anatomi dan mikromorfologi daun mempunyai nilai taksonomi yang boleh digunakan dalam pengecaman spesies bagi genus *Thunbergia* khususnya pada *T. erecta* dan *T. laurifolia.*

**Highlight**

- Five common characteristics of *Thunbergia erecta* and *Thunbergia laurifolia* were the presence of raphide, sinuous anticlinal walls, diacytic stomata, majority opened and minority closed venation in lamina and the presence of peltate glandular (unicellular terminal) trichome.
- Several variable characteristics to distinguish *Thunbergia erecta* and *Thunbergia laurifolia* were petiole and marginal outlines, types of vascular bundles, the presence of druse, marginal venation, stomata occurrence, types of wax, cuticular sculpturing and types of trichomes.
- Leaf anatomy and micromorphology are proven to be additional tools to provide useful information for the identification of *Thunbergia* species.

## INTRODUCTION

The Acanthaceae family is one of the plant families that belong to the order Lamiales. According to Wasshausen and Wood (2004), the Acanthaceae is a large and diverse pantropical family consist of approximately 240 genera and 3,250 species. Recently, the systematic position of Acanthaceae is divided into four subfamilies which are; Acanthoideae, Nelsonioideae, Thunbergioideae and Avicennioideae (Stevens 2016). The Acanthaceae is mainly herbaceous and shrubs but sometimes found as climbers or lianas especially in the genus *Thunbergia*, while rarely found as woody plants or trees (Metcalfe & Chalk 1965; Carlquist 1988; Scotland *et al*. 1995; Vollesen 2008). One of the most important criteria in recognising the Acanthaceae is the occurrence of cystoliths. However, Heywood *et al.* (2007) reported on the absence of cystoliths in the vegetative parts of subfamilies Nelsonioideae, Thunbergioideae and the tribe of Acanthae.

*Thunbergia* Retz. is a large genus of the Acanthaceae subfamily Thunbergioideae (Takhtajan 1997). The genus *Thunbergia* is comprised of more than 100 species (Chia-chi *et al*. 2011) distributed in the tropical and subtropical regions of Africa, Madagascar, Asia and Australia (Borg *et al.* 2008). Kar *et al*. (2013) explained that *Thunbergia* was named in 1780 by Retzius, in the honours of Carl Peter Thunberg (1743–1828), a Swedish botanist, doctor and naturalist. Most of the *Thunbergia* species are known as clock vine which referred to its clockwise twinning habit (Retief & Reyneke 1984). The Asia *Thunbergia* is taxonomically characterised by a few morphological characters such as perennial herbaceous or woody climbers, shrubs, rarely erect or trailing herbs without the occurrence of cystoliths (Suwanphakdee & Vajridaya 2018). To be noticed, many of the *Thunbergia* species preferred full sun exposure and well-drained soil but somehow can bloom in the partial shady areas (Sultana *et al*. 2015).

Moreover, the leaves are usually simple, petiolate, opposite arranged, ovate or lanceolate to hastate or sagitate shaped and the margins are entire to lobed to dentate. Borg *et al.* (2008) also mentioned that this genus is differed from other members of the family by their reduced calyx and enlarged bracteoles. Apart, seeds of the Acanthaceae are usually borne on hook-like retinacula that attached to the septa of the capsules but sometimes lacking in *Thunbergia* (Chia-chi *et al.* 2011). The present research thereby deals with two species of *Thunbergia* namely; *Thunbergia erecta* (Benth.) T. Anderson and *Thunbergia laurifolia* Lindl., which can be differentiated obviously by the characteristics of their floral part. *T. erecta* showed a solitary type of inflorescence with purplish-blue corolla, whereas *T. laurifolia* as in raceme inflorescence with blue-violet corolla. However, taxonomists might face difficulty to identify the species if the absence of the floral part since both *Thunbergia* species studied shared similarities in the vegetative parts. Even Nurul-Aini *et al.* (2018) mentioned that the identification and classification of the Acanthaceae species is quite challenging due to morphological similarities shared with other species in the same genus. Leaf anatomy and micromorphology hence are very useful tools to provide the additional data for the identification purposes as well to support the taxonomic study of the species, especially when the absence of floral and fruiting materials (Khatijah & Ruzi 2006).

According to Sultana *et al.* (2015), most of the *Thunbergia* species possessed the ornamental values because of their attractive flowers and twinning characters. Previous studies also reported on the utilisation of certain *Thunbergia* species in India for medicinal purposes. For instance, *Thunbergia grandiflora* has been useful in treating minor eyesore and against snake bite as reported by Teron (2005), whereby Nath and Dutta (2010) explained on the uses of *T. grandiflora* stem juice against conjunctivitis among the indigenous tribe. Also, Sarma (2006) reported on the usage of *Thunbergia coccinea* roots against dysentery, stomach ache and fever. Additionally, Aritajat *et al.* (2004) reported on the usage of *T. laurifolia* in treating diabetes among Thai traditional healers. Besides, the herbal teas and capsules of *T. laurifolia* are produced and marketed by the local companies in Thailand (Chan *et al.* 2011). The growing interest in the potential medicinal sources from the genus *Thunbergia* in India and Thailand is considered as a good starting point for pharmacological research. However, the lack of attempt is reported on the potential medicinal sources from the genus *Thunbergia* in Peninsular Malaysia. Therefore, this research is expected to be a good platform to encourage more discoveries on the potential medicinal plants in Peninsular Malaysia. In fact, the present comparative study is significant to avoid misidentification of *Thunbergia* species that might lead to incorrect harvesting of raw materials and drug authentication, especially to produce medicines. Thereby, this present study is conducted to investigate the comparative leaf anatomy and micromorphology of two selected *Thunbergia* species; *T. erecta* and *T. laurifolia* in Peninsular Malaysia, and later determine whether they possessed taxonomic values that might be useful in the identification and classification of *Thunbergia*, especially at the species level. Also, this research aims to contribute to the knowledge and documentation of these selected *Thunbergia* species from Peninsular Malaysia.

## MATERIALS AND METHODS

This study was carried out on two selected taxa from the genus *Thunbergia* of Acanthaceae subfamily Thunbergioideae which were; *T. erecta* and *T. laurifolia*. Fresh specimens were obtained from several forest reserves in Peninsular Malaysia such as in Hutan Lipur Lata Tembakah, Besut, Terengganu and Hutan Lipur Lata Kinjang, Perak. Three replicates of each plant species were collected throughout this research. The voucher specimens were deposited at Universiti Kebangsaan Malaysia Herbarium (UKMB) for future reference. Fresh specimens collected were fixed in 3:1 AA solutions (70% Alcohol: 30% Acetic Acid). For the leaf anatomical methods, part of petioles, midribs, leaf lamina and marginal were sectioned in a range of thickness (30 μm–40 μm) using sliding microtomes. The epidermal peels were conducted manually by scrapping off the underside of leaf surfaces by using a razor blade. The leaf clearing involved the immersion of leaf lamina and margin into the Basic Fuchsin solution. All the anatomical procedures including the staining and dehydration process followed the suitable modifications methods by Johansen (1940) and Saas (1958). Anatomical images were captured using a video (3CCD) camera attached to a Leitz Diaplan microscope using Cell^B software. For the leaf micromorphology method, the specimens were obtained from dried samples of herbarium. Samples lamina were cut about 1 cm^2^. The specimens were then mounted on a mounting holder and coated with gold by using a sputter-coated machine. Micromorphological images were captured by using a scanning electron microscope Philips XL Series XL 30 with magnifications of 150×, 300×, 700× and 1,000×.

## RESULTS AND DISCUSSIONS

**Figure 1:**
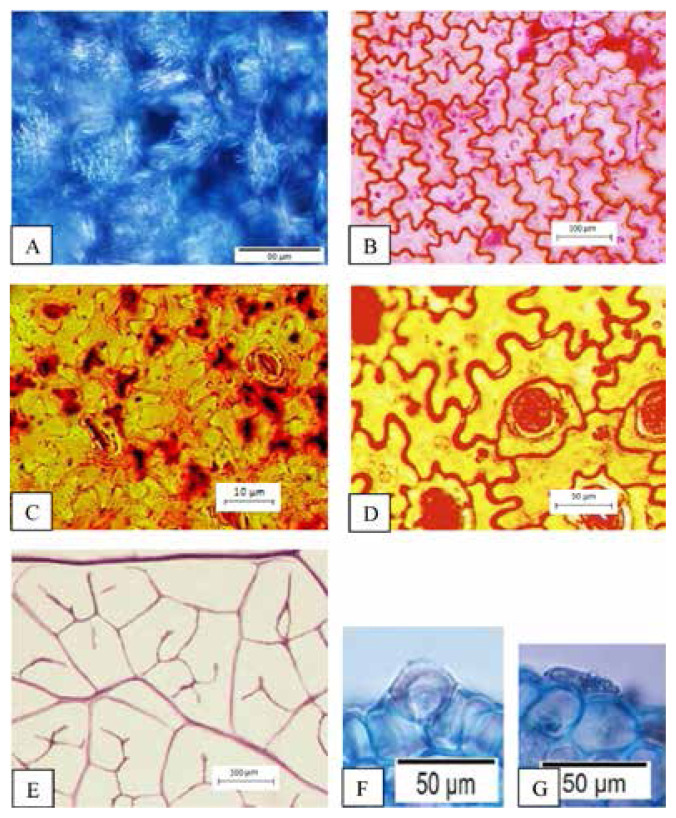
Common characteristics of leaf anatomy and micromorphology in *Thunbergia* (A) Presence of raphide, (B) Sinuous anticlinal walls on adaxial surface, (C) Sinuous anticlinal walls on abaxial surface, (D) Presence of diacytic stomata, (E) Lamina venation with majority opened and minority closed with swollen tracheid, (F and G) Presence of peltate glandular trichomes (unicellular terminal). Scales: (E) 500 μm; (B) 100 μm; (A, D, F and G) 50 μm; (C) 10 μm.

The findings of this study showed the presence of five common characters of leaf anatomy and micromorphology among two selected *Thunbergia* species. The common characteristics are the presence of raphide in the parenchyma cortex of petiole and midrib (Fig. 1A), sinuous anticlinal walls on adaxial and abaxial surfaces (Fig. 1B), presence of diacytic stomata (Figs. 1C and 1D), lamina venation with majority opened and minority closed with swollen tracheids (Fig. 1E) and presence of peltate glandular trichomes (unicellular terminal) (Figs. 1F and 1G).

**Figure 2:**
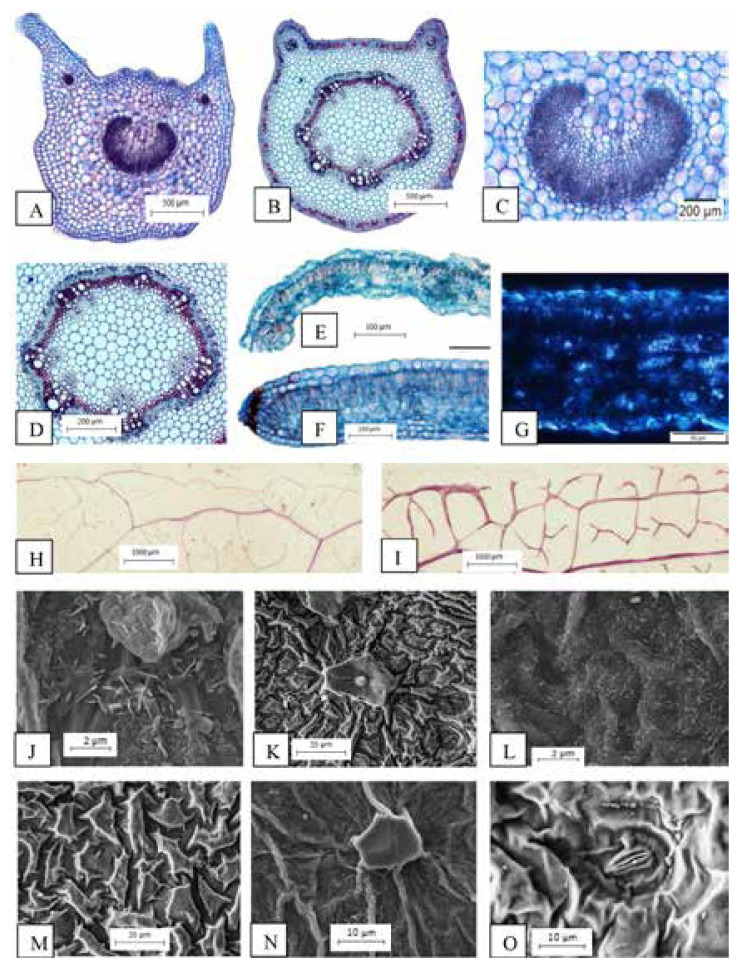
Variation characteristics of leaf anatomy and micromorphology in *Thunbergia* (A and B) Petioles outlines (C) Opened vascular bundles, (D) Closed vascular bundles, (E) Marginal outline; round shaped with 90° curved, (F) Marginal outline; round shaped and straight, (G) Presence of druses, (H) Complete marginal venation, (I) Incomplete marginal venation, (J) Types of wax; verrucate, flakes and granules, (K) Types of wax; crustose and granules, (L) Cuticular ornamentation; anticlinal and periclinal walls can be slightly differentiated, (M) Cuticular ornamentation; Sinked anticlinal walls; raised periclinal walls, (N) Stomata absent on adaxial surface (hypostomatic) and (O) Stomata present on adaxial surface (amphistomatic). *Scales*: (H and I) 1000 μm; (A and B) 500 μm; (C, D and F) 200 μm; (E) 100 μm; (G) 50 μm; (K and M) 20 μm; (N and O) 10 μm; (J and L) 2 μm.

The variation characteristics examined are petiole outlines; horns-like shape at the right and left upper corners of adaxial surface with U-shaped of abaxial surface in *T. erecta* (Fig. 2A). While in *T. laurifolia*, lobes at the right and left upper corners of adaxial surface and flat in the middle of adaxial surface with ¾ round shaped of abaxial surface (Fig. 2B). Besides, the main vascular bundle (opened system with continuous ring of vascular bundle) and additional vascular bundles (opened system with two continuous rings of vascular bundles) are recorded in petiole of *T. erecta* (Fig. 2C). Whereas main vascular bundle (closed system with continuous ring of vascular bundle) and additional vascular bundles (opened system with two continuous rings of vascular bundle) are observed in petiole of *T. laurifolia* (Fig. 2D). For marginal outlines; round-shaped with 90° curved in *T. erecta* (Fig. 2E) whilst round shaped and straight in *T. laurifolia* (Fig. 2F). The presence of druses also observed in lamina part of *T. erecta* (Fig. 2G). For marginal venation; complete venation is recorded in *T. erecta* (Fig. 2H) whereas incomplete venation in *T. laurifolia* (Fig. 2I). Four types of waxes present in this study which are; verrucate, flakes and granules observed in *T. erecta* (Fig. 2J) while crustose and granules in *T. laurifolia* (Fig. 2K). Anticlinal and periclinal walls can be slightly differentiated in *T. erecta* (Fig. 2L) but sinked anticlinal walls and raised periclinal walls are recorded for *T. laurifolia* (Fig. 2M). Stomata are absent on adaxial surface of *T. erecta* (hypostomatic) (Fig. 2N) yet present on adaxial surface of *T. laurifolia* (amphistomatic) (Fig. 2O).

Candolle (1879) described on the several fundamental concepts of petiole anatomy and distinguished between two systems of vascular bundles which are; open system and close system of vascular bundles. Even Hare (1942) emphasised on two types of vascular tissue existed in petiole which are open system (U-shaped) and close system (O-shaped). Previous studies on the petiole anatomy of some Philippines *Shorea* species also recognised the taxonomic distinction in petiole vascular patterns (Rojo 1987). Not only that, few studies affirmed that petiole anatomy can be used for grouping genera and identification of species (Agbagwa & Ndukwa 2004; Noraini & Cutler 2009). In fact, the petiole anatomy can be used to form groups in the genus *Dipterocarpus* (Ruzi *et al.* 2009). Recently, Nurul-Aini *et al.* (2018) also agreed on the significance of vascular bundles types in taxonomic study as in three selected Acanthaceae species studied, namely *Acanthus ebracteatus* (Vahl), *Andrographis paniculata* (Burm.f.) Wall. Ex Nees and *Chroesthes longifolia* (Wight) B. Hansen. The importance of midrib vascular bundles characterisctics also proven to be useful to identify and differentiate the genus and species as in Rubiaceae (Nurul-Syahirah *et al.* 2016). The present study hence reported on the main vascular bundle (opened system with continuous ring of vascular bundle) and additional vascular bundle (opened system with two continuous rings of vascular bundles) are recorded in petiole of *T. erecta*, whereas the main vascular bundle (closed system with continuous ring of vascular bundle) and additional vascular bundles (opened system with two continuous rings of vascular bundles) in *T. laurifolia*. Therefore, the character of vascular bundles might be useful and provide additional data to differentiate between these two species.

Recently, the leaf anatomy study conducted on selected Acanthaceae taxa in Peninsular Malaysia showed several characteristics that can be used in the identification of Acanthaceae including petiole and midrib outlines (Nurul-Aini *et al.* 2018). This present study hence proved that *Thunbergia* studied also can be distinguished by the petiole and marginal outlines. Petiole outlines in *T. erecta* appeared as a horns-like shape at right and left upper corners of adaxial surface; U-shaped of abaxial surface, whereby lobes at right and left upper corners and flat in the middle of adaxial surface; ¾ round-shaped of abaxial surface in *T. laurifolia*. The marginal outlines in *T. erecta* occurred in round-shaped with 90° curved, whereas round-shaped and straight in *T. laurifolia*. Findings on the taxonomic value of the petiole and marginal outlines in *Thunbergia* species also supported by Nurul-Aini *et al*. (2013) in the classification species from the genus *Microcos* (Tiliaceae) by using petiole outlines criteria.

Beck (2010) explained that protoplast consists of two major categories of waste metabolites which are; calcium oxalate crystals and tanniferous substances. According to Franceschi and Nakata (2005), the presence of these small crystals in higher plants is common and usually related to the physical protection, storage of calcium, removal of oxalate from the metabolic system and regulation of light during photosynthesis in plants that grow under shade. Noraini *et al.* (2019) even stressed that the occurrence of ergastic substances such as calcium oxalate and calcium carbonate crystals are usually related to the physiological activities of the plants. There are two typical types of crystals found in plants known as druse and raphide. The compact and spherical aggregates of angular crystals are known as druse, whereas the aggregations of needle-like crystal known as raphide (Beck 2010). However, the occurrence of crystals, distributions, or types of prismatic either druse or raphide can be used as a taxonomic character, especially for species identification. Metcalfe and Chalk (1985) mentioned the presence of raphide, druse, styloid, prismatic and sand-like crystal idioblasts in species of Rubiaceae. The presence of druse only in the lamina part of *T. laurifolia*, and raphide in the parenchyma cortex of *Thunbergia* studied thereby suggests that this might be a significant characteristic for the genus and also to differentiate these two species.

According to Cutler *et al.* (1978), the characters on leaf epidermis can be used as a diagnostic feature in the identification of plant species. Fahn (1967) explained that many of the epidermal cells appeared in tabular form by which mostly sinuous anticlinal walls occurred in the leaf blade of dicotyledons, whereby elongated in monocotyledons. Moreover, the value of the sinuous wall is reported to increase the tensile strength of the epidermis. For instance, the geometric features of the sinuous shape of *Dryopteris thelypteris* L. are reported to provide a more secure attachment between the epidermal and mesophyll cells (Korn 1976). The present study of *Thunbergia* species has recorded on the sinuous anticlinal walls of abaxial and adaxial surfaces, thus considered as a common character for the genus. Apart, the results of leaf surface studies also reported on the occurrence of hypostomatic leaves in *T. erecta* whereas amphistomatic leaves in *T. laurifolia*. Fahn (1967) explained that hypostomatic leaves referred to the presence of stomata only on the upper surface of leaves, whereby stomata present on both sides of leaves are known as amphistomatic. The basis of leaf epidermal structure is important for species identification as reported by Nurul-Aini *et al.* (2014) on the presence of hypostomatic and amphistomatic leaves among eight chosen taxa of Acanthaceae. Even Robbrecht (1988) mentioned that hypostomatic leaves are common in Rubiaceae. Therefore, results on the occurrence of stomata are considered as important criteria to distinguish *Thunbergia* species. Not only that, Metcalfe and Chalk (1950) categorised the dicotyledons stomata into four main types based upon the basis arrangement of the subsidiary cells which are; anomocytic, anisocytic, paracytic and diacytic. Recently, Noraini *et al.* (2019) divided the stomatal types in plants into 10 categories which are; polocytic, amphidiacytic, diallelocytic, tetracytic, staurocytic and cyclocytic. The present leaf epidermal study on *Thunbergia* hence might confirm that the diacytic stomata found in both species studied are common in *Thunbergia* species as reported by Ahmad (1974).

Hickey (1973) claimed that the criteria of leaf venation is related to plant evolution and possessed as an important taxonomic value in plant systematic. The main functions of leaves including light-harvesting, gaseous exchange, water transport, and photosynthate distribution depend on the architectural elements that are related to the arrangement of veins or called a venation pattern. Ummu-Hani *et al.* (2014) classified two types of ultimate marginal venation in the genus *Ficus* (Moraceae) which are; incomplete ultimate marginal venation (freely ending veinlets directly towards adjacent of the margin) and complete ultimate marginal venation (higher vein orders fused into a vein running just inside the margin). Furthermore, Sehgal and Paliwal (2008) also mentioned that anatomical studies on the ornamentation of leaf veins have significant values for the identification of *Euphorbia* species. Therefore, the complete marginal venation in *T. erecta* and incomplete venation in *T. laurifolia* can be used as a distinctive character to distinguish these two species. The present study also reported on the lamina venation with majority opened and minority closed with swollen tracheids in both *Thunbergia* studied, hence might be considered as a common character for the genus.

Leaf surface investigations have been more focussed compared to other plant surfaces because it comprised of variable features that occurred within taxa and useful in taxonomic applications. The results of the leaf surface studies are reported to be useful in the examination of genera and species of various families such as Podocarpaceae (Mill & Schilling 2009) and Rubiaceae (Moraes *et al.* 2009). Even Barthlott (1998) affirmed that the epicuticular wax structures possessed a considerable ultrastructural and chemical diversity, and significant in taxonomic study. The waxes have ecological importance especially for the interaction between the environment and plants. The appearance of verrucate, flakes and granules cuticular wax of *T. erecta*, and crustose and granules cuticular wax in *T. laurifolia* proved that the cuticular wax can be one of the important characters to distinguish these two species. The significance of the cuticular wax character in *Thunbergia* studied is supported by Nurul-Aini *et al.* (2014) on the recognition of six types of epicuticular wax found in eight chosen taxa of Acanthaceae. Furthermore, Nurhanim *et al.* (2014) also mentioned that cuticular sculpturing is a criterion that can be used to distinguish plant species in *Schoutenia* Korth. (Malvaceae). There are three types of cuticular sculpturing that have been reported in five selected species of *Schoutenia.* The present study however reported that cuticular sculpturing can be slightly differentiated in *T. erecta*, whereas appeared as sinked anticlinal walls and raised periclinal walls in *T. laurifolia*. Somehow, the micromorphological aspects of the leaf surface are influenced by its habitat (Dunn *et al.* 1965). Moraes *et al.* (2011) also mentioned that cuticular ornamentation in some of *Psychotria* species does not appear to occur because of the sunlight exposure factor as the individuals grow in the understory of the forest.

Werker (2000) defined trichomes as unicellular or multicellular appendages that originated from the epidermal cells and develop outwards on the surface of various plant organs. Also, Navarro and El-Oualidi (2000) stated that trichomes are hairs which commonly can be found on the epidermis of plants. To be noticed, the trichomes morphology served an important taxonomic character and useful in the plant systematic. Beiras and Sajo (2009) also used the types of trichomes for the identification and classification of woody plants in Brazilian savannah. In fact, trichomes morphology is not only served in the identification of species but also important in pharmacognosy, archaeobotany, paleobotany and agronomy (Rao & Ramayana 1977). Apart, Kim *et al.* (2012) mentioned that trichomes also act as a physical and chemical barrier against biotic and abiotic stresses. For convenience purposes, the following structures of trichomes in *Thunbergia* studied are categorised into six recognisable types in Table 1. The types of trichomes are illustrated by drawings to avoid complex descriptions. Findings of the study recognised the five types of trichomes found in *T. erecta* which are; Types 1, 2, 3, 5 and 6, compared to *T. laurifolia* which consists of only two types of trichomes; Types 1 and 4. Moreover, the common type of trichomes found in *Thunbergia* studied is peltate glandular (unicellular terminal) trichome (Type 1). The criteria of trichomes morphology is proven to be useful to distinguish both species of *Thunbergia* studied, hence supported by previous research on the significance of trichomes morphology on petals of some Acanthaceae species (Amirul-Aiman *et al.* 2014).

## CONCLUSION

In conclusion, this study indicates that leaf anatomy and micromorphology characters are important in the taxonomic study, especially to distinguish some species in *Thunbergia*. Findings from this study showed five typical characters occurred in *T. erecta* and *T. laurifolia* including the presence of raphide in the parenchyma cortex of petiole and midrib, sinuous anticlinal walls on abaxial and adaxial surfaces, presence of diacytic stomata, lamina venation with majority opened and minority closed with swollen tracheids and presence of peltate glandular (unicellular terminal) trichomes. These common characteristics can be used as taxonomic criteria in characterise species from the genus *Thunbergia*. Furthermore, the absence of cystoliths in *Thunbergia* studied confirms that subfamily Thunbergioideae does not possess cystoliths, hence can be a significant taxonomic criterion to recognise species from the genus *Thunbergia*. Results from this study also revealed several interesting features, with that might be useful to distinguish *T. erecta* and *T. laurifolia*. The variable characteristics examined are petioles and marginal outlines, types of vascular bundles, the presence of druses in the lamina part, types of marginal venation, cuticular wax, cuticular sculpturing, stomata occurrence and types of trichome. This study confirms that leaf anatomy and micromorphology characteristics possessed taxonomic importance and act as supportive evidence in the identification and classification of plants either at species or genus level.

## Figures and Tables

**Table 1:** Types of trichomes studied.

Type	Trichomes descriptions	Pictures	Illustrations
Type 1	Peltate glandular (unicellular terminal)	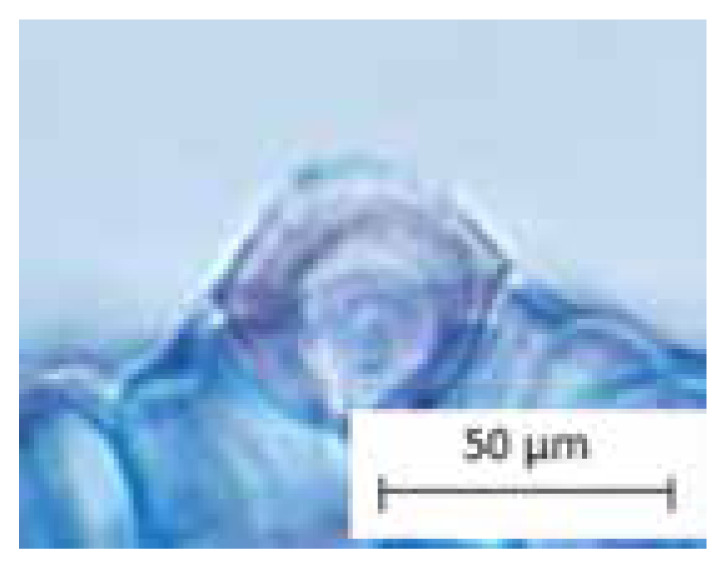	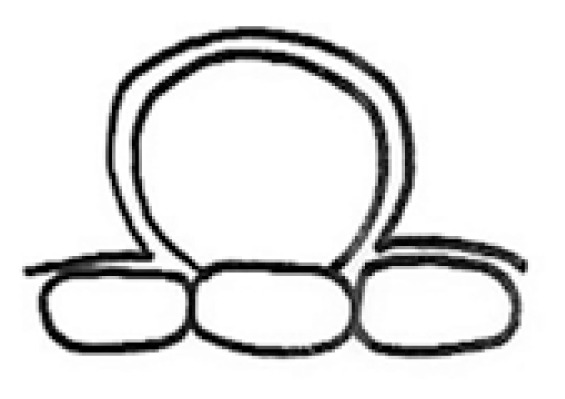
Type 2	Peltate glandular (multicellular terminal)	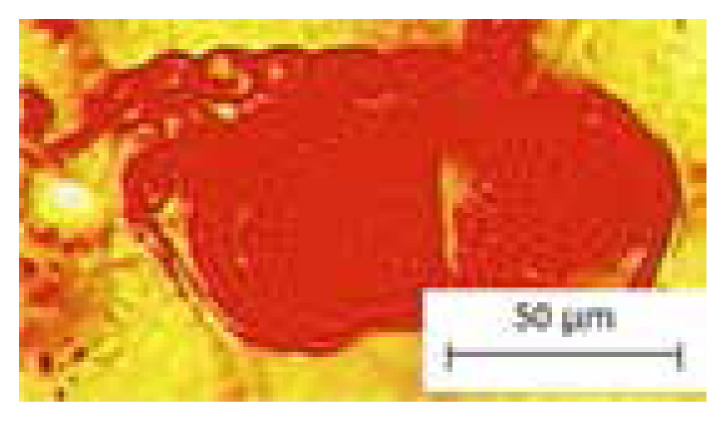	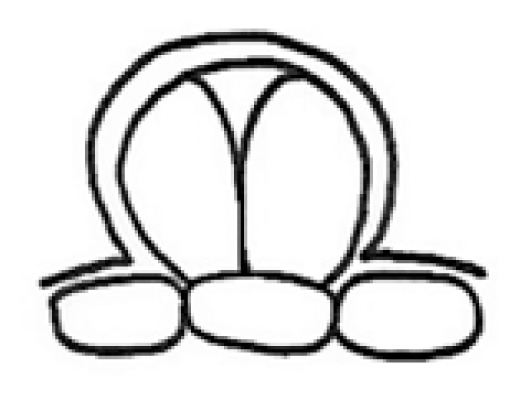
Type 3	Simple unicellular (short, blunt end)	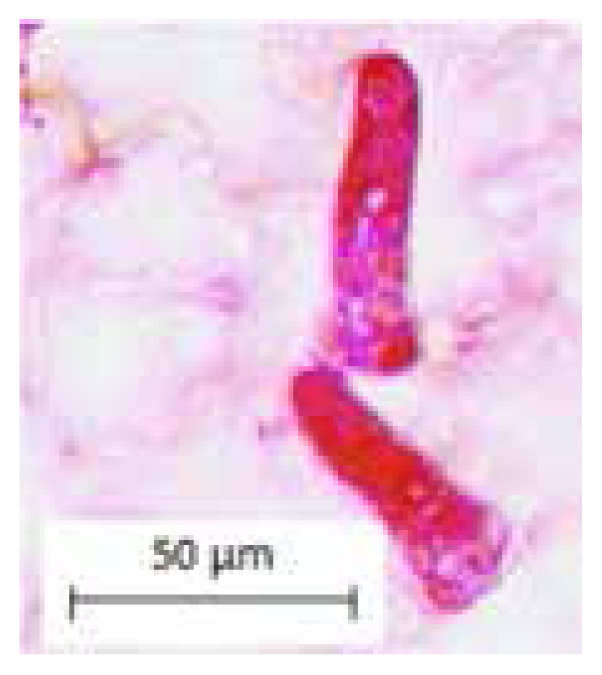	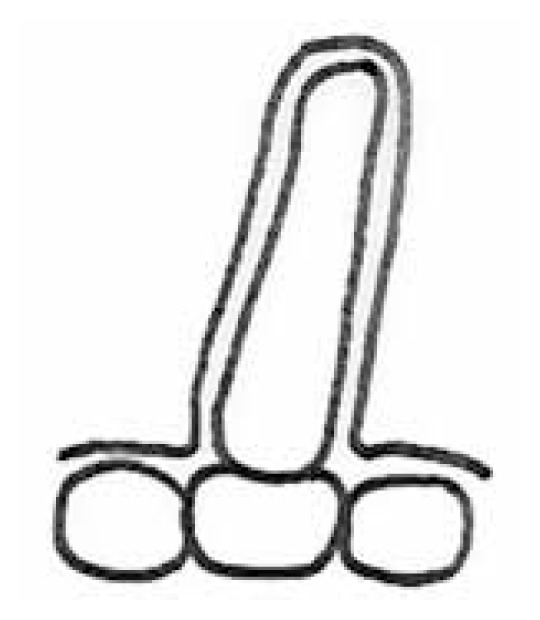
Type 4	Simple unicellular (short, pointed end)	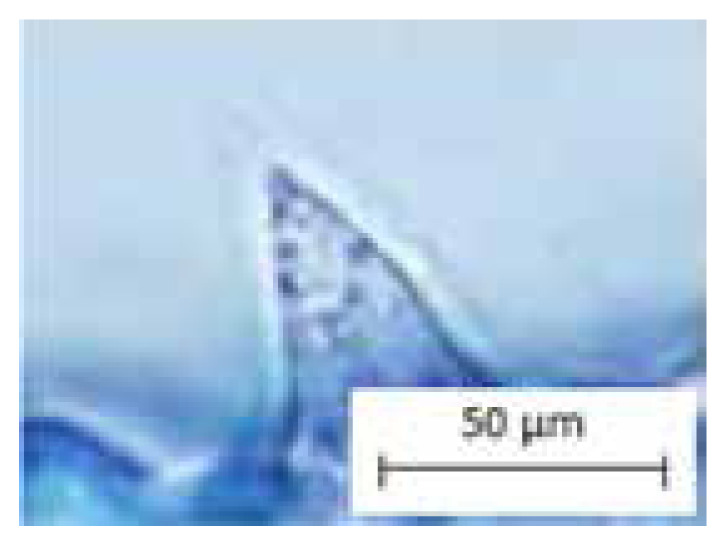	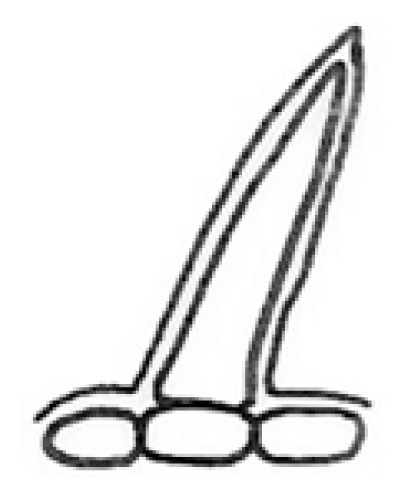
Type 5	Simple multicellular (short, blunt end)	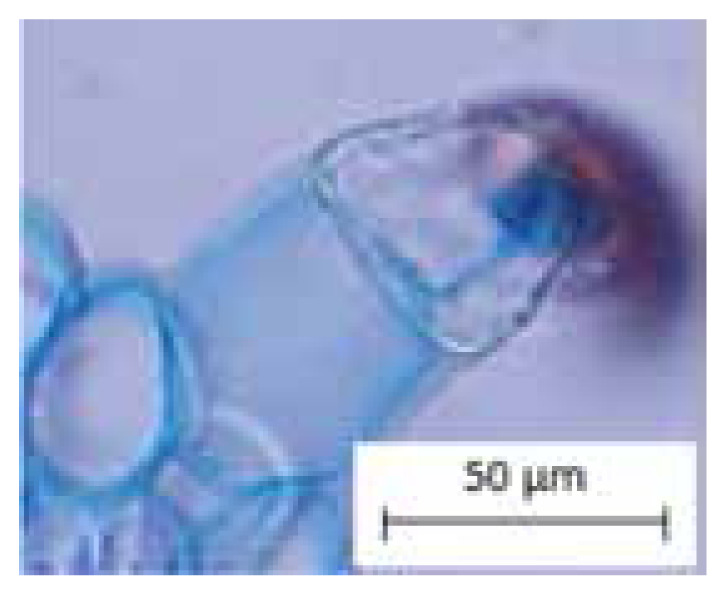	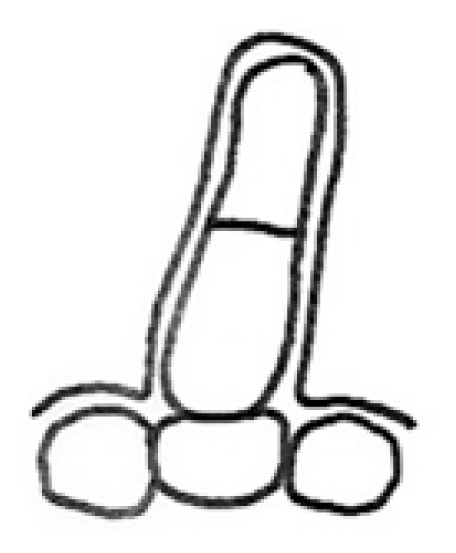
Type 6	Simple multicellular (short, pointed end, echinate)